# A Practical Do-It-Yourself Recruitment Framework for Concurrent eHealth Clinical Trials: Identification of Efficient and Cost-Effective Methods for Decision Making (Part 2)

**DOI:** 10.2196/11050

**Published:** 2018-11-29

**Authors:** Emily G Lattie, Susan M Kaiser, Nameyeh Alam, Kathryn N Tomasino, Elizabeth Sargent, Caryn Kseniya Rubanovich, Hannah L Palac, David C Mohr

**Affiliations:** 1 Center for Behavioral Intervention Technologies Department of Medical Social Sciences Northwestern University Feinberg School of Medicine Chicago, IL United States; 2 Center for Behavioral Intervention Technologies Department of Preventive Medicine Northwestern University Feinberg School of Medicine Chicago, IL United States; 3 AbbVie Inc North Chicago, IL United States

**Keywords:** eHealth, mHealth, mental health, recruitment

## Abstract

**Background:**

The ability to successfully recruit participants for electronic health (eHealth) clinical trials is largely dependent on the use of efficient and effective recruitment strategies. Determining which types of recruitment strategies to use presents a challenge for many researchers.

**Objective:**

The aim of this study was to present an analysis of the time-efficiency and cost-effectiveness of recruitment strategies for eHealth clinical trials, and it describes a framework for cost-effective trial recruitment.

**Methods:**

Participants were recruited for one of 5 eHealth trials of interventions for common mental health conditions. A multipronged recruitment approach was used, including digital (eg, social media and Craigslist), research registry-based, print (eg, flyers and posters on public transportation), clinic-based (eg, a general internal medicine clinic within an academic medical center and a large nonprofit health care organization), a market research recruitment firm, and traditional media strategies (eg, newspaper and television coverage in response to press releases). The time costs and fees for each recruitment method were calculated, and the participant yield on recruitment costs was calculated by dividing the number of enrolled participants by the total cost for each method.

**Results:**

A total of 777 participants were enrolled across all trials. Digital recruitment strategies yielded the largest number of participants across the 5 clinical trials and represented 34.0% (264/777) of the total enrolled participants. Registry-based recruitment strategies were in second place by enrolling 28.0% (217/777) of the total enrolled participants across trials. Research registry-based recruitment had a relatively high conversion rate from potential participants who contacted our center for being screened to be enrolled, and it was also the most cost-effective for enrolling participants in this set of clinical trials with a total cost per person enrolled at US $8.99.

**Conclusions:**

On the basis of these results, a framework is proposed for participant recruitment. To make decisions on initiating and maintaining different types of recruitment strategies, the resources available and requirements of the research study (or studies) need to be carefully examined.

## Introduction

### Background

Recruiting participants into electronic health (eHealth) intervention efficacy trials has long been a challenge [1,2]. Although internet access has become increasingly widespread and the digital divide has narrowed in recent years [3], difficulties remain in reaching individuals who are both representative of the target population and interested in taking part in these trials [4,5]. There are ever increasing ways of recruiting, from older, more traditional methods such as mailing or public print advertising, to newer methods such as social media, and resources such as registries and marketing firms, and each method comes with a set of costs and benefits.

In recent years, difficulties associated with developing and testing new eHealth programs under traditional research grant timelines have been identified [6-8]. Given the focus of the National Institute of Mental Health on information technologies for social and behavioral health [9] and the increase in health researchers who are now capitalizing upon the widespread adoption of personal technologies in attempts to expand the reach and accessibility of behavioral interventions, it is increasingly important that researchers choose efficient recruitment strategies to maximize their research funds and timelines and hit recruitment targets to allow for robust evaluation of program quality, efficacy, and effectiveness. Time and costs required to design and program technologies, as well as the unanticipated, albeit inevitable development problems, often squeeze out time and resources intended for trial recruitment. Although past reviews [10,11] have highlighted the value of using Facebook and other social media methods for health research recruitment, there have been few studies reporting on the efficiency of these recruitment methods relative to other recruitment methods for health intervention research. Thus, there remains a need to examine the costs and benefits of multiple methods of recruitment to identify those methods that are likely to be efficient and cost-effective.

### Objectives

The Center for Behavioral Intervention Technologies (CBITs) at Northwestern University recently completed enrollment for 5 simultaneous clinical trials of eHealth interventions for common mental health conditions (ie, depression and anxiety). To support this enrollment effort, CBITs developed a clinical trial recruitment support system [12] and a set of recruitment methods that were flexible to the target populations required for each of the trials. This paper presents descriptive information regarding the recruitment strategies employed by CBITs during a nationwide recruitment for eHealth clinical trials, the efficiency of these strategies in producing referred and enrolled participants, and the estimated cost of using these strategies. Given the diverse set of responsibilities needed to successfully employ these strategies, we provide a description of the roles and relevant expertise of our research study staff.

The aim of this paper was to propose a decision framework for cost-effective trial recruitment. To support this aim, we describe a set of procedures that were used to recruit and enroll participants across 5 trials using a *do-it-yourself* (DIY) recruitment support framework described in the companion paper [12]. We then analyze cost-effectiveness of the recruitment strategies used. Finally, these data, along with lessons learned, are used to propose a framework for recruitment decision making.

## Methods

### Study Descriptions

During the recruitment period reported on in this paper, we conducted 3 trials for adults older than 18 years and 2 trials for targeted age groups (ie, high school students, adults aged 65 years and older), all of which evaluated eHealth interventions for the treatment or prevention of common mental health conditions (ie, depression, anxiety) and included a national recruitment strategy. The companion paper by Palac et al [12] also includes a trial that was conducted exclusively in the Chicago area.

The trials for adults older than 18 years are described below:

#### Stepped Care Randomized Controlled Trial

The Stepped Care randomized controlled trial (RCT) recruited adults older than 18 years who were currently experiencing a depressive episode. Through random assignment, the study compared up to 20 weeks of (1) a telephone-administered cognitive behavioral therapy (T-CBT) and (2) a stepped care intervention that initiated treatment with a coached internet CBT program called ThinkFeelDo, stepping those participants who did not show improvement up to T-CBT (outcome paper currently under review). Follow-up assessments were administered by phone and Web-based questionnaire up to 2 times during the 20-week treatment period and at 3 and 6 months post treatment.

#### IntelliCare Field Trial

The IntelliCare Field Trial evaluated a suite of 13 Android apps with adults older than 18 years with symptoms of anxiety, depression or both [13]. Of these, 12 apps provided different clinical therapy skills for treating anxiety and/or depression, and 1 app, named the IntelliCare Hub, served as a central place to manage the other apps. All participants used the apps for 8 weeks and were provided with access to a coach via SMS text messaging (short message service, SMS). Participants completed Web-based questionnaires assessing symptom change and provided user feedback about the apps at 4 and 8 weeks into the study.

#### IntelliCare Randomized Controlled Trial

This RCT continued the evaluation of the IntelliCare platform using a 2×2 factorial design in which participants were randomized to receive (1) coaching or no coaching and (2) automatic weekly recommendations versus no automated recommendations [14]. Participants were asked to use the apps for 8 weeks, completing 2 Web-based questionnaires during the active app use study period, and again 3 and 6 months after the end of the 8-week active app use period (primary outcome paper is currently in preparation).

The 2 trials for targeted age groups, which both utilized a group social networking component, are described below:

#### ProjectTECH Field Trial

ProjectTECH tested an online and Web-app based group intervention for the prevention of teenage depression and substance use disorders [15]. Youth in the age group of 14 to 19 years were placed into peer groups and provided an adapted, responsive version of ThinkFeelDo that was available on phones with age-appropriate content and was embedded in an activity feed that supported communication among group members. The peer groups were facilitated by either a clinical psychologist or a high school student peer guide. Participants were asked to use the Web platform for 8 weeks. They were sent online questionnaires at 4, 8, and 12 weeks after beginning the study.

#### MoodTech Field Trial

MoodTech adapted the ThinkFeelDo program for the treatment of depression among adults older than 65 years [16]. All users had the support of the same clinical psychologist to coach them on how to use the website. Participants were assigned to 1 of 3 groups and either had access to a version of the website they could use independently, a version of the website that included peer support features and as well as an online space where they could interact with a small group of their peers or they were assigned to a wait list control group. Both versions of treatment were 8 weeks long. Follow-up assessments were administered by phone and online questionnaires at 4, 8, and 12 weeks after starting to use the website. Participants placed on the wait list before using the website; they completed 2 additional assessments during the waiting period and then had access to the independent version of the site.

### Recruitment Strategies

Participants were recruited for these trials using a multipronged approach, including digital (eg, social media and Craigslist), research registry-based, print (eg, flyers and posters on public transportation), clinic-based (eg, a general internal medicine clinic within an academic medical center and a large nonprofit health care organization), a market research recruitment firm, and traditional media strategies (eg, newspaper and television coverage in response to press releases). Participants self-reported their recruitment source on an initial online screening survey, and recruitment source was clarified when contact was made with study staff.

For each recruitment strategy, the research team prepared verbiage (and in some cases images) with target populations in mind. All verbiage and images were approved by the institutional review board (IRB) before use and all online advertisements directed to full study information on a separate website and/or the online study prescreen survey. An example of these recruitment advertisements can be seen in Multimedia Appendix 1.

For social media ads including Facebook, Instagram, and Twitter, a bank of IRB-approved verbiage and images was used to create brief teaser ads. This allowed staff members posting ads to mix and match the headlines, body text, and associated photos of advertisements, thus avoiding repetitive content. Messages were targeted toward the group of interest for each study (eg, an older adult sample, a teen sample, and a general adult sample) and on criteria including but not limited to geographic location, gender, and those who had indicated interest in any number of relevant keywords such as “depression” and “anxiety.” We typically ran social media ads for a month at a time but were more interested in how the ads were performing while adhering to budgetary constraints. Once an ad set started running, we would let it run for several days to 1 week before checking in the progress. Ads that were not performing well were turned off, and resources were reallocated to better performing ads. We typically used the standard delivery type (which spreads out the ads throughout a selected campaign schedule); however, when we were trying to recruit quickly, we would turn on the accelerated delivery type.

For research registry-based recruitment effort, invitations were crafted for various research registries with verbiage directed toward individuals interested in helping further research knowledge. With some registries such as ResearchMatch, the research team could target invitations based on age, race/ethnicity, previous diagnoses reported, and area of residence in the United States.

For print-based recruitment, flyers and posters were designed by research staff members and printed through companies that had partnerships with the research team’s university. Flyers were placed in various businesses with community boards such as coffee shops and on various university boards in common areas and in medical office waiting rooms with medical staff permission. Research staff utilized their university’s partnership with the Chicago Transit Authority to purchase flyer space on buses and trains at a discounted rate. Train and bus routes that were generally busy and ran close to the university were chosen for advertising.

For clinic-based recruitment, research staff partnered with physicians to refer patients by providing information about the studies. For 1 study, the research team partnered with a large nonprofit health care organization that orchestrated referrals from their clinics.

For market research recruitment firm-based recruitment, the research team worked with a research recruitment firm that was able to recruit interested volunteers from across the United States. Email invitations were first sent internally by the research recruitment firm to participant panelists. The email contained a link to a study screener that was adapted for and hosted on the market research firm’s site. Research staff had to develop unique recruitment verbiage and a separate online screener for individuals from the market research firm.

For traditional media strategy-based recruitment, the research team’s university media relations department typically wrote and released an article about results of studies previously conducted within our research center and provided contact information (eg, email, website, and phone) for those interested in enrolling in an ongoing study. The research team would then receive calls or emails from interested participants. Once an online prescreening system was implemented, interested volunteers were directed to the research website and online prescreening link.

### Staff Roles and Expertise

Recruitment efforts were conducted under the leadership of an MPH-level research manager (SMK) with experience in community mental health and clinical trial management. This individual managed a team of research staff for the clinical trials unit (CTU), which was composed of bachelor’s and master’s level staff. A total of 23 individuals supported study recruitment over the recruitment period, including 3 staff members from Northwestern Clinical and Translational Sciences Institute who specialized in study screening and were brought on when our team exceeded capacity to manage recruitment and clinical interviews. At the peak of recruitment, there was a core team of 10 CTU staff members supporting recruitment efforts. Most of the research staff members had primary roles as clinical interviewers or technology support specialists on these trials and managed specific recruitment strategies as a smaller component of their work week. For a 6-month period, a digital marketing manager worked with the CTU on developing a robust social media strategy focused on Instagram, Facebook, and Twitter.

The recruitment strategies mentioned above directed participants to a centralized online screening survey to be prescreened for the center’s actively recruiting clinical trials. The online survey was used to automate initial eligibility decisions, eliminating individuals who would be ineligible for all studies, and allowing research assistants more time to interact with potentially eligible participants and confirm eligibility via a brief phone screener.

A master’s level data manager, experienced in programming language for data wrangling, managed the back-end automation and routing of potential participants from various recruitment sources through this centralized online screening survey. For the back-end automation, programming code was written to automatically screen participants for entry into the center’s active clinical trials and route to a study based on specific study eligibility, participants’ preferred study choice, and the center’s recruitment targets for each active study. Code was updated as new recruitment sources were added and center recruitment targets changed. New referrals were processed daily and based on the number of eligible participants received. The data manager notified team members to increase or decrease recruitment efforts, particularly on digital strategies such as social media ads and research registry pulls. This is described in further detail in the study by Palac and colleagues [12] and was a critical and cost-efficient contributor to the success of the recruitment strategies described in this paper. As the data component of the framework described by Palac et al [12] was based on technologies already supported by our university (and commonly found at other universities), there were no additional technology costs to maintain this support system.

### Recruitment Process

Potential participants could contact the center via email, telephone, our Web screening survey, or from an in-app interest form. The IntelliCare apps were publicly available on the Google Play Store [17], and people who had already downloaded an IntelliCare app could complete a form within the app that indicated their interest in participating in relevant research projects. These potential participants are labeled as “contacted” throughout this paper. Then, all potential participants went through a brief screening measure and, if initially eligible, were phone-screened by a research assistant. These potential participants are labeled as “screened” throughout this paper. Finally, eligible potential participants who passed the 2-stage screening and enrolled in 1 of the 5 clinical trials described above are labeled as “enrolled” throughout this paper.

### Data Analysis

Descriptive statistics were computed to characterize (1) the number of potential participants labeled as “contacted,” (2) the number of those potential participants labeled as “screened,” and (3) the number of participants from each recruitment source labeled as “enrolled.” To highlight differences between the intervention trials included in this study, the demographics of participants and the number of enrolled participants from each recruitment source by trial were also computed. Per-participant costs were calculated for each recruitment method based on a ratio of participant yield to expenditures. The time costs of each recruitment method were calculated based on objective review of study records (eg, meeting minutes) and through estimates made in consultation with study staff regarding the time study staff members spent on the launch and maintenance of each research strategy while it was being utilized. Time estimates were then converted to time costs by multiplying hours spent by the relevant hourly wage (eg, US $17.50 for research assistant time and US $30.17 for research manager time). Fees for each of the recruitment methods were calculated based on billing records. Then, the participant yield on recruitment costs was calculated by dividing the number of enrolled participants by the total cost for each method. This analytic method allows for the identification of methods that were particularly cost efficient and time efficient for recruiting eligible participants, while providing transparency into the inner and outer system fees associated with each set of recruitment methods. Results from these analyses were then used to outline a framework for recruitment decision making in the Discussion section.

## Results

As shown in Table 1, there was considerable variability in the staff skills and time required to establish and maintain the recruitment strategy.

**Table 1:** Summary of recruitment strategies

Recruitment strategy	Digital	Registry	Print	Clinic	Firm	Media
Topperforming sites	Instagram, Reddit, Craigslist	ResearchMatch	Ads on Chicago Transit Authority bus and train lines	Health partners, Group Health	Focus Pointe Global	Unable to be determined
Techniques	Social media marketing, content marketing, direct email, eNewsletters, app advertising, study description on website, blog posts	Email (direct and through Web portal) to registry participants	Approximately 800 study-specific banner ads were placed on 2 of Chicago’s busiest train lines and on 18 bus routes.	Invitation mailed via United States Postal Service, email invitation sent via electronic medical record portal, phone call from research assistant	Email invitation through firm	Planned press release, reprints
Target population	US general public (adults and adolescents)	Registry participants (adults)	Chicago general public (adults)	Individuals engaged in care systems (adults)	Market research firm panelists (adults)	US general public (adults)
Staff skills required for startup/management	Social media marketing, analytics, design, public relations (crisis response), REDCap [18]	Human subjects recruitment	Design	Project management, relationship management, stakeholder management, database management, human subjects recruitment	Project management, database management, clinical trials recruitment	Public relations, journalism
Management effort	Daily management	Weekly management	Monthly management	Weekly management	Weekly management	As needed (but labor intensive during initial media blitz)
Resource considerations	Nearly infinite in terms of reaching new potential participants	Finite number of registry participants	Cost prohibitive. University discount made it possible to advertise broadly	Finite number of patients	Finite number of participants	Nearly infinite in terms of reaching new potential participants

**Table 2:** Potential participants by recruitment source.

Recruitment strategy		Digital	Registry	Print	Clinic	Firm	Media	Other	Unknown	Unknown (IntelliCare) app/ Web form	Total
**Raw numbers (n)**											
	Contacted	3318	2030	789	3261	290	297	33	472	6727	17,217
	Screened	895	627	308	266	138	144	9	33	86	2506
	Enrolled	271	225	89	75	55	49	3	6	4	777
**Outcomes (%)**											
	Percent of patients contacted that were screened	26.97	30.89	39.04	8.16	47.59	48.48	27.27	6.99	1.28	—^a^
	Percent of paitents contacted that were enrolled	8.17	11.08	11.28	2.30	18.97	16.5	9.09	1.27	0.06	—
	Percent of patients screened that were enrolled	30.28	35.89	28.9	28.2	39.86	34.03	33.33	18.18	4.65	—

^a^Not applicable.

A total of 17,217 potential participants contacted the recruitment site, 2506 completed screening, and 777 were enrolled across the studies. Table 2 displays that the number of potential participants from each recruitment source who had contact with our research center during the trial enrollment period varied greatly. The largest portion of potential participants came from an unknown source (ie, the recruitment source was missing from their record, usually because of the participant’s failure to respond to that query) and had contacted the research center through the IntelliCare in-app interest form (labeled IC app/Web form in Table 2). This means that these potential participants had already downloaded an IntelliCare app, but we do not know how they first learned about the IntelliCare suite of apps. Among potential participants from a known source, the majority came from digital recruitment strategies (eg, social media and Craigslist), followed by clinic-based recruitment (eg, a general internal medicine clinic in an academic medical center and a large nonprofit health care organization), research registries (eg, ResearchMatch website), print-based advertising (eg, flyers and posters on public transportation), and media (eg, news stories prompted by press releases from our research center that included information about ongoing trial recruitment). The smallest portion of participants were recruited from “other” sources, which included recruitment sources such as being referred to our center by another research lab and learning about our center through public events.

The Outcomes section of Table 2 shows that the potential participants who contacted our center and failed to identify how they arrived at the site (both the “unknown” and the “unknown IC app/Web form”) had extremely low rates of screening completion (<7% for the general unknown category and <2% for those who contacted us through the IntelliCare in-app interest form), while those who identified how they arrived at the site had substantially better rates of Web-screening completion, ranging from 8% for clinic-based recruitment to 48% for media-based recruitment and market research firms, with digital recruitment strategies yielding 27%. The strategies that yielded the highest rates of conversion from contact to screening were the use of a market research recruitment firm (48%) and the use of research registries (31%), both of which target individuals who are likely to be interested in research participation. Overall, digital recruitment strategies yielded the largest number of participants across the 5 clinical trials, with nearly 35% of the total enrolled participants coming in from digital recruitment strategies. Registry-based recruitment strategies were in second place by enrolling nearly 29% of the total enrolled participants across trials.

To highlight differences in the use and success of recruitment strategies for the different targeted trials, Table 3 presents the number of participants enrolled by each recruitment source by trial.

As seen in Table 4, enrolled participants were similar to the overall demographic of United States and largely representative of individuals seeking mental health treatment in the United States in that there was overrepresentation of women and non-Hispanic white individuals.

Table 5 displays the fees and time costs per person screened, and cost per person enrolled varied considerably across recruitment strategies. During the recruitment period for the 5 clinical trials included in this paper, a total of US $144,537.67 were spent on recruitment fees, and there was a total estimated time cost of US $19,834.59 for a combined total of US $164,372.26. The fees, which included those fees that were paid to enact and maintain the recruitment strategies, ranged from US $1 per person enrolled for research registry-based recruitment to US $1,218.33 per person enrolled for clinic-based recruitment. The time costs, or research staff hourly wages required to implement and maintain the recruitment strategies, ranged from US $8.99 per person enrolled for research registry-based recruitment to US $75.01 per person enrolled for clinic based recruitment.

Research registry-based recruitment had particularly low fees (eg, many registries were free to post in, and nominal fees amounted to US $150 total) and had an associated moderate time cost. As research registry-based recruitment had a relatively high conversion rate from potential participants who contacted our center to be screened to be enrolled, registries have presented as the most cost-effective method for enrolling participants in this set of clinical trials, with a total cost per person enrolled at US $8.99. However, these registries are typically a finite resource. As recruitment progressed, the research team exhausted the supply of registry participants such that the registries were not accumulating new potentially eligible participants at a rate that kept up with recruitment needs.

**Table 3:** Participants enrolled by recruitment source.

Name of trial	Stepped Care RCT, n (%)	IntelliCare RCT, n (%)	IntelliCare Field Trial, n (%)	ProjectTech Field Trial, n (%)	MoodTech Field Trial, n (%)
Digital	111 (35.6)	103 (34.2)	25 (23.8)	30 (76.9)	8 (17.0)
Registry	99 (31.7)	72 (23.9)	17 (16.2)	0 (0)	35 (74.5)
Print	35 (11.2)	41 (13.6)	10 (9.5)	0 (0)	3 (6.4)
Clinic	35 (11.2)	0 (0)	39 (37.1)	0 (0)	1 (2.1)
Firm	0 (0)	55 (18.3)	0 (0)	0 (0)	0 (0)
Media	17 (5.4)	25 (8.3)	7 (6.7)	0 (0)	0 (0)
Other	13 (4.2)	5 (1.7)	3 (2.9)	9 (23.1)	0 (0)
Unknown/in-app referral	2 (0.6)	0 (0)	4 (3.8)	0 (0)	0 (0)

**Table 4:** Participant demographics by trial.

Demographics		Stepped Care RCT^a^ (N=312)	IntelliCare RCT (N=301)	IntelliCare Field Trial (N=105)	ProjectTECH Field Trial (N=39)	MoodTech Field Trial (N=47)
Age in years, mean (SD)		37.7 (14.2)	36.5 (11.8)	38.9 (14.1)	16.23 (0.99)	69.6 (4.1)
**Gender, n (%)**						
	Female	229 (73.4)	228 (75.7)	80 (76.2)	29 (74)	32 (68)
	Male	81 (26.0)	71 (23.6)	25 (23.8)	9 (23)	15 (31)
	Other	2 ( 0.6)	2 (0.7)	0 (0)	1 (3)	0 (0)
**Race, n (%)**						
	American Indian or Alaska Native	0 (0)	0 (0)	1(1)	0 (0)	0 (0)
	White	275 (88.1)	237 (78.7)	88 (83.8)	24 (62)	41 (87)
	African American	21 ( 6.7)	29 (9.6)	8 (7.6)	3 (8)	2 (4)
	Asian	14 ( 4.5)	10 (3.3)	6 (5.7)	4 (10)	0 (0)
	More than one race	8 (2.6)	18 (6.0)	1 (1)	4 (10)	3 (6)
	Unknown/declined to report	0 (0)	7 (2.3)	1 (1)	4 (10)	1 (2)
**Ethnicity, n (%)**						
	Hispanic or Latino	32 (10.3)	30 (9.9)	5 (4.8)	10 (26)	1 (2)
	Not Hispanic or Latino	275 (88.1)	268 (89.0)	99 (94.3)	29 (74)	46 (98)
	Hispanic or Latino—unknown or not reported	5 (1.6)	3 (1.0)	1 (1)	0 (0)	0 (0)

^a^RCT: randomized controlled trial.

**Table 5:** Fees and time costs for recruitment strategies (in USD).

Recruitment strategy		Digital	Registry	Print	Clinic	Firm	Media	Total
**Fees**		$11,726.01	$150.00	$9318.66	$91,375.00	$31,968.00	$0.00	$144,537.67
	Fees per person screened	$13.10	$0.24	$30.26	$343.52	$231.65	$0.00	
	Fees per person enrolled	$43.27	$1	$104.70	$1218.33	$581.24	$0.00	
**Time cost**		$8601.25	$1872.50	$761.53	$5767.52	$1896.52	$935.27	$19,834.59
	Time cost per person screened	$9.61	$2.99	$2.47	$21.68	$13.74	$6.49	
	Time cost per person enrolled	$31.74	$8.32	$8.56	$76.90	$34.48	$19.09	
**Total cost**		$20,327.26	$2022.50	$10,080.19	$97,142.52	$33,864.52	$935.27	$164,372.26
	Total cost per person screened	$22.71	$3.23	$32.73	$365.20	$245.40	$6.49	
	Total cost per person enrolled	$75.01	$8.99	$113.26	$1295.23	$615.72	$19.09	

## Discussion

### Principal Findings

Results from this set of 5 eHealth intervention trials focused on common mental health problems (ie, depression and anxiety) indicate that use of digital recruitment strategies (eg, Facebook, Instagram, and Craigslist) and research registry-based recruitment strategies (eg, ResearchMatch) were the most fruitful, time-efficient, and cost-effective methods for recruiting a nationwide sample of participants who were largely representative of the populations of interest. These results add to the literature on clinical trial recruitment methods and the benefits of technology-enabled recruitment strategies. Findings are partially consistent with systematic review results recently reported by Whitaker et al [10] on the topic of using Facebook to recruit participants for health research purposes. Whitaker et al [10] found growing evidence that, when compared with traditional recruitment methods (eg, print, radio, and email), Facebook recruitment had multiple benefits including lower costs and shorter recruitment periods. However, that review only included 1 study focused on mental health and did not examine the utility of other digital recruitment methods such as Instagram and Craigslist. These results also partially support findings of a scoping review by Topolovec-Vranic and Natarajan [11] in which digital recruitment strategies (eg, Facebook and Craigslist) were compared with other recruitment strategies for medical research study recruitment. Of the 30 studies included in their review, 12 studies found that digital strategies were more effective than other methods, and an additional 3 studies found that digital strategies were equally effective as another recruitment strategy. However, only 10 of the 30 studies were on behavioral interventions, and none of them were on eHealth interventions for common mental health problems. Although these studies provide support for the use of digital strategies for medical/health-related study recruitment, they do not reflect the unique nature of recruiting participants with common mental health problems for eHealth interventions. Thus, the results presented in this paper contribute to the broader literature by honing in on this population for eHealth intervention research and by examining additional recruitment strategies (eg, Instagram and ResearchMatch).

Results of analyses, combined with research staff experiences, have been used to develop a framework for recruitment strategy decision making for eHealth interventions depicted in the questions to guide strategic decision making presented in Table 6 and the matrix of recruitment strategy benefits presented in Table 7. In Table 7, we have highlighted the recruitment strategies that offer primary benefits of low fees, a high degree of control over the number and flow of referrals being directed to research staff, access to large numbers of people, access to targeted populations (eg, with specific clinical diagnoses and with specific demographic profiles), and 2 benefits associated with easier management/maintenance of the recruitment strategy (ie, a lack of specialized skills needed and a relatively low burden/time effort for study staff).

Using a variety of recruitment strategies is recommended, and the tools presented in Tables 6 and 7 are intended to help researchers determine the best subset of strategies to use for a particular study or set of studies. To efficiently manage multiple strategies, we recommend implementing a recruitment support framework as described by Palac et al [12], which is structured around an online screening survey and a central tracking database overseen by a data manager. To make decisions on initiating and maintaining different types of recruitment strategies, careful examination of the resources available (ie, budget, staff, relationships, and discounts) and requirements of the research study (ie, target recruitment number, target participant flow/timeline, and entry criteria) is essential. However, before reviewing the Table 6 question set and Table 7 matrix to determine one’s optimal recruitment strategies, one should conduct a literature review to determine if there are relevant studies that suggest what the outcomes or conversion rates for screening to enrollment could be for one’s target population using recruitment strategies that may already be under consideration. Early identification of conversion rate estimates for screening to enrollment will help the research team make appropriate time-cost and fee-related investments from the beginning of a trial. If there are no estimates available, then researchers will need to experiment with their selected set of recruitment strategies to fine-tune their approach.

Throughout the question set in Table 6, one is prompted to consider the existing resources and requirements for a specific study. These resources include available funds (ie, the budget), staff expertise, staff effort, existing relationships, and access to discounts. As our research center was concurrently recruiting for multiple clinical trials, we were afforded some flexibility using recruitment funds to test multiple recruitment strategies and to start and stop the use of those strategies as needed.

**Table 6:** Questions to guide strategic decision making for recruitment.

Topics		Questions
**Resources**		
	Budget	Do you have a budget for paid advertising? Do you have a budget to support staff to manage the strategy?
	Staff expertise	Can you recruit or train staff to learn skills required to set up/manage this strategy?
	Staff effort	Do you have staff who will be available to establish/manage this strategy?
	Relationships	Do you have relationships to establish this strategy?
	Discounts	Do you have or can you make connections to reduce the overall cost of this strategy?
**Requirements**		
	Target (N)	How many people do you need to recruit overall (<100, >100)?
	Flow/timeline	How quickly do you need to enroll subjects (months, years)? Do you have enough time to experiment?
	Entry criteria	How stringent are your entry criteria (ie, how targeted do you need to be with your advertising?)

**Table 7:** Matrix of recruitment strategy benefits.

Benefits	Digital	Registry	Print	Clinic	Firm	Media
Low fees		✓				✓
High degree of control (can control number and flow of referrals)	✓	✓			✓	
Broad reach (access large numbers of people)	✓				✓	✓
Access to a targeted population	✓			✓		
No specialized skills required for maintenance/management		✓	✓			✓
Low effort required for maintenance/management		✓	✓			

Furthermore, most research staff members had a primary role as a clinical interviewer or as a technology support specialist on these trials and managed specific recruitment strategies as a smaller component of their work week. As research staff employed in a primary capacity for clinical interviewing typically had times during the workday in which no interviews were taking place, there was bandwidth to develop specialized skills and to manage more time-intensive recruitment strategies. Thus, the capacity for recruitment strategies requiring specialized skills (such as digital, clinic, and firm-based strategies) and higher levels of effort for management (such as those needed to maintain digital, clinic, firm, and media-based strategies) was already built into the structure of the research team. As seen in Table 6, study requirements include the target sample size, the target flow/timeline of participants getting screened and enrolled in the study, and the study’s entry criteria, which can all be assessed to determine which recruitment strategies are most likely to be fruitful. Studies requiring a large sample size will need to utilize strategies capable of tapping into large numbers of potential participants, and for studies that have a limited timeline for recruitment, it will be important to pick a few recruitment strategies and monitor their success closely so that the research team can adjust the strategies as needed. Studies with stringent entry criteria need to be more targeted in their advertising (relative to studies that are recruiting a general adult sample), and this can increase the fees associated with certain types of recruitment (eg, online advertisements) and increase the time necessary to develop and design appropriate recruitment advertisements.

As identified in our results, the cost-effectiveness and time-efficiency of the recruitment strategies employed varied significantly, with digital and registry-based recruitment strategies demonstrating the greatest degree of cost-effectiveness and time-efficiency. This was likely because of the ability of our research team to control the number and flow of referrals using these 2 strategies, and thus, we were able to get large numbers of potentially eligible participants into our studies in a relatively efficient manner. However, many of the costs presented in this paper are dependent on multiple factors and thus can be estimated differently based on resources available in different research settings. For example, the expertise that staff members already possess (eg, social media expertise) can contribute to certain recruitment efforts (eg, digital strategies) in ways that reduce the need for hiring outside consultants or contractors. Alternately, a lack of these types of internal expertise would not preclude a research team from undertaking these types of recruitment strategies but could increase the costs of engaging in these strategies, as it may be a less efficient use of a staff member’s time. Similarly, the existing state of relationships with clinics and health care systems can dramatically impact the time costs and fees associated with clinic-based recruitment. Building new relationships takes significant time, and strong existing relationships may come with reduced fees within certain clinics and health care systems.

Furthermore, recruitment-associated fees can vary depending on existing institutional relationships and access to support such as discounts. For example, the price that our research center paid for recruitment advertisements on public transportation was at a reduced cost because of an arrangement previously established by our Northwestern University’s Clinical and Translational Sciences Institute with the public transportation service. Recruitment-associated fees can also vary depending on changing advertising fee structures and the popularity of keywords used in the advertisements [19,11]. One recent systematic review on the cost of recruiting for research studies using Facebook found that researchers paid between US $1.36 and US $110 per completing participant [20]. Although the majority of studies (80%) included in this review were cross-sectional surveys, and, thus, those ad clicks were more likely to convert to active study participation compared with intervention studies that last several weeks to months, findings by Thornton et al [20] demonstrate the broad range of fees that can be applied to use of a single digital recruitment strategy.

For research studies with a limited staff that are targeting fee-related cost-efficiency, reliance on registry-based and media-based strategies as primary recruitment efforts could prove to be both realistic and successful to hit recruitment targets, provided that the research registries utilized include a feasible number of potential participants (see Table 7). Print strategies may also be considered for these cases if the research team is able to locate low-cost print outlets that are likely to reach their target population. The use of digital recruitment strategies (eg, Facebook, Twitter, and Instagram) can also be feasible for studies with limited staff if the study team contains at least one individual with a firm understanding of digital marketing, or if there is support for a study team member to develop this expertise. The use of these strategies requires initial management decisions (eg, reliance on paid ad campaigns vs time developing more robust but free Web presence) but can be designed to require less staff time than was used by our group while still allowing researchers to draw from a very large number of potential participants and exert a high degree of control over the flow of potential participants from targeted populations.

Our personnel cost estimates are pulled from a private university in a large Midwestern city and may not accurately reflect pay rates in other areas of the United States or in other cities around the world conducting similar research. Indeed, clinical research costs are largely driven by personnel costs, and these costs can be substantially lower or higher in other locations where similar research could feasibly take place [21]. Some researchers may struggle with personnel-related decisions because of financial costs, and we note that having an experienced research manager can be more costly upfront but has the potential to save money over time because of skill at managing other research staff time and at negotiating relationships with new recruitment partners. This was particularly important during the set of trials used in this paper, as our experienced research manager was key in negotiating and navigating relationships and keeping recruitment targets on track to ensure that money was being well spent. This tracking system is further described in our companion piece by Palac et al [12].

We found that digital and research registry-based recruitment strategies brought in a faster flow of participants than other strategies examined and that this can be particularly useful for studies with a limited recruitment timeline. This is partially consistent with past review papers on using social media for research recruitment [10,11]. Although digital strategies can be designed to tap into a growing audience through slight shifts in targeting, strategies such as clinics and research registries may limit recruitment efforts as they tend to have a relatively fixed number of potential participants. Not surprisingly, the recruitment strategies used and their relative success varied by target population. Although recruitment using digital and research-registry based strategies were similarly successful in our studies of general adult samples, some differences were noted in our studies focused on specific age groups, as seen in Table 3. In our ProjectTECH study of high school students [15], the vast majority of participants were recruited through Instagram, a social media platform that was particularly popular with teenagers during the recruitment period. In the MoodTech study for older adults [16], recruitment via digital platforms was less successful, and the vast majority of participants were recruited through the ResearchMatch registry.

To our knowledge, the time-efficiency and cost-effectiveness of research registry-based recruitment for eHealth interventions has not previously been reported upon and compared with other methods of recruitment such as digital strategies and more traditional strategies such as clinic-based recruitment and print advertisements. Results of this study suggest that, as the most cost-effective method of recruitment that also yielded a high percentage of eligible participants, researchers should strongly consider strategies such as the ResearchMatch registry to identify individuals who are likely to be interested and eligible for their eHealth intervention studies. The use of research registries appears to be far more efficient and inexpensive compared with print advertisements, recruitment firms, and clinic-based strategies. However, given that research registries are typically drawing from a finite group of potential participants, the use of supplementary recruitment strategies is valuable.

### Research Considerations

An issue that emerges here is the denominator problem, as previously discussed by Mohr et al [22]. The denominator problem notes that most eHealth interventions recruit from very large pools of potential participants, and thus those individuals who choose to participate in an eHealth program are likely uniquely motivated. Although this paper focuses on recruitment for early efficacy trials of eHealth programs, we note that the time-efficient and cost-effective recruitment strategies discussed in this paper may further contribute with regard to testing eHealth interventions on the select group of individuals in the general population who are likely to engage and benefit from these interventions. A broader use of recruitment strategies produces the possibility of a wider range of participants, but this does not necessarily solve the denominator problem. As the goal of eHealth program development is to ultimately have the potential for larger scale implementation and public health benefits, an exclusive focus on recruiting for efficacy trials is likely to have a detrimental impact on the potential for developing programs to be successfully implemented. Thus, researchers may be wise to consider at least preliminary assessment of implementation factors in early evaluations of eHealth interventions following the guidelines for type 1 hybrid trials described by Curran et al [23].

Although findings indicate that clinic-based recruitment strategies were expensive and inefficient in this set of trials, we do not conclude that researchers avoid partnerships with clinical care settings when evaluating eHealth interventions for common mental health problems. Rather, the data presented here demonstrate that digital and research-registry recruitment strategies are efficient and relatively inexpensive for enrolling participants in these types of studies. For researchers focused on bringing their eHealth programs into clinical practice settings, the additional time and effort needed to enroll participants from a clinical practice setting is vital and will come with valuable insights into barriers and facililators to larger scale program implementation. To maximize time-efficiency and cost- effectiveness, the strategies described in this paper should be used in tandem with clinical trial recruitment support systems focused on prescreening referral (as described by Palac et al [12]).

### Limitations

The examination of recruitment strategy efficiency and cost-effectiveness and the resulting decision-making framework presented in Tables 6 and 7 are not without limitations. This was based on a limited number of clinical trials of eHealth interventions for common mental health conditions. However, the recruitment principles listed within this paper are likely generalizable to clinical trials focusing other types of digital behavior change and health interventions. Furthermore, the time spent on various recruitment efforts was not closely tracked during these trials, and thus the time costs of many strategies were estimated through a combination of objective review of study records (eg, meeting minutes) and through estimates made in consultation with study staff regarding the time study staff members spent on the launch and maintenance of each research strategy while it was being utilized.

Another limitation of this study is the large percentage of potential participants who came from unknown sources and after completing an in-app research interest form, did not proceed with screening. Media-based recruitment, in which press releases from our research center included information about ongoing trial recruitment, initially appear to be a relatively low-cost recruitment strategy. Yet, many of these “unknown” participants contacted our research center following periods of media coverage, and although we can hypothesize that a sizeable portion of these individuals learned about our trials and downloaded 1 or more IntelliCare apps through media coverage, we cannot substantiate this hypothesis. Although it is clear that media coverage generated a small stream of referrals who went on to complete screening and enroll in the study, the influx of potential participants (many of which are labeled as being from unknown sources) contacting our research staff following press releases required a fairly high management effort by study staff.

This decision-making framework is less relevant if it is important for an intervention to be tested within a specific clinic. In those cases, the recruitment strategies will have to be focused within the clinic, and recruitment timelines and budgets will have to be established to account for a potentially slow recruitment speed/low recruitment yield and to account for what could be substantial time costs and fees associated with establishing the clinic relationship, navigating acceptable referral methods, and advertising to clinic patients (eg, mailing study advertisments to all potentially eligible patients within clinic can be high cost and low yield, while personnel time required to conduct daily chart review and identify potentially eligible participants to approach may be more fruitful). These barriers to quick recruitment in clinic settings have been well documented previously and must be planned around [24,25]. For a review of best practices in study site selection and recommendations to plan efficient recruitment efforts in these clinical contexts, see Huang et al’s’ recent paper on the Clinical Trials Transformation Initiative [26]. More commonly, eHealth interventions are being tested for efficacy and can draw from a broader pool of potential participants. In these cases, the framework can be used to evaluate the resources available and the requirements (ie, main aims and constraints) of the study.

Despite these weaknesses, in tandem with the system described by Palac et al [12], this is the first framework for designing and monitoring recruitment efforts for eHealth clinical trials. This framework can be used by fellow researchers to make recruitment decisions at the outset of an eHealth clinical trial to target a set of efficient and cost-effective recruitment efforts and can be used as recruitment needs and priorities may shift over the course of a clinical trial.

### Conclusions

In our study, digital and research registry-based recruitment strategies are more efficient and cost-effective for engaging potential participants in trials evaluating eHealth interventions aimed at common mental health problems (ie, depression and anxiety) when compared with traditional recruitment strategies such as print-based advertisements and recruitment from within clinical care systems. These results also demonstrate how a DIY recruitment framework can be used to track recruitment success and cost-effectiveness and support recruitment strategy decision making. These methods, along with the topics proposed in the recruitment strategy framework, should be considered by researchers when designing their recruitment strategies, with specific focus on the overarching aims of the study (eg, getting participants in quickly to test an intervention, compared with focusing on how an intervention would fit into a specific clinical care setting).

## Appendix

Multimedia Appendix 1 Examples of recruitment advertisements used for various recruitment sites.

## Abbreviations

CBITs: Center for Behavioral Intervention Technologies
CTU: clinical trials unit
DIY: do-it-yourself
eHealth: electronic health
IRB: institutional review board
RCT: randomized controlled trial
T-CBT: telephone-administered cognitive behavioral therapy

## References

[1] Thompson D, Canada A, Bhatt R, Davis J, Plesko L, Baranowski T, Cullen K, Zakeri I (2006). eHealth recruitment challenges. Eval Program Plann.

[2] Ahern DK, Patrick K, Phalen JM, Neiley JD (2006). An introduction to methodological challenges in the evaluation of eHealth research: perspectives from the Health e-Technologies Initiative. Eval Program Plann.

[3] Smith A (2017). Pew Research.

[4] O'Connor S, Hanlon P, O'Donnell CA, Garcia S, Glanville J, Mair FS (2016). Understanding factors affecting patient and public engagement and recruitment to digital health interventions: a systematic review of qualitative studies. BMC Med Inform Decis Mak.

[5] Nelson LA, Zamora-Kapoor A (2016). Challenges in conducting mHealth research with underserved populations: lessons learned. J Telemed Telecare.

[6] Muessig KE, Nekkanti M, Bauermeister J, Bull S, Hightow-Weidman LB (2015). A systematic review of recent smartphone, Internet and Web 2.0 interventions to address the HIV continuum of care. Curr HIV/AIDS Rep.

[7] Wu YP, Steele RG, Connelly MA, Palermo TM, Ritterband LM (2014). Commentary: pediatric eHealth interventions: common challenges during development, implementation, and dissemination. J Pediatr Psychol.

[8] Pagliari C (2007). Design and evaluation in eHealth: challenges and implications for an interdisciplinary field. J Med Internet Res.

[9] National Institute of Mental Health (2017). Opportunities and Challenges of Developing Information Technologies on Behavioral and Social Science Clinical Research.

[10] Whitaker C, Stevelink S, Fear N (2017). The use of Facebook in recruiting participants for health research purposes: a systematic review. J Med Internet Res.

[11] Topolovec-Vranic J, Natarajan K (2016). The use of social media in recruitment for medical research studies: a scoping review. J Med Internet Res.

[12] Palac HL, Alam Nameyeh, Kaiser Susan M, Ciolino Jody D, Lattie Emily G, Mohr David C (2018). A Practical Do-It-Yourself Recruitment Framework for Concurrent eHealth Clinical Trials: Simple Architecture (Part 1). J Med Internet Res.

[13] Mohr D, Tomasino KN, Lattie EG, Palac HL, Kwasny MJ, Weingardt K, Karr CJ, Kaiser SM, Rossom RC, Bardsley LR, Caccamo L, Stiles-Shields C, Schueller SM (2017). IntelliCare: an eclectic, skills-based app suite for the treatment of depression and anxiety. J Med Internet Res.

[14] Cheung K, Ling W, Karr CJ, Weingardt K, Schueller SM, Mohr DC (2018). Evaluation of a recommender app for apps for the treatment of depression and anxiety: an analysis of longitudinal user engagement. J Am Med Inform Assoc.

[15] Lattie EG, Ho J, Sargent E, Tomasino KN, Smith JD, Brown CH, Mohr DC (2017). Teens engaged in collaborative health: the feasibility and acceptability of an online skill-building intervention for adolescents at risk for depression. Internet Interv.

[16] Tomasino KN, Lattie EG, Ho J, Palac HL, Kaiser SM, Mohr DC (2017). Harnessing peer support in an online intervention for older adults with depression. Am J Geriatr Psychiatry.

[17] Lattie E, Schueller SM, Sargent E, Stiles-Shields C, Tomasino KN, Corden ME, Begale M, Karr CJ, Mohr DC (2016). Uptake and usage of IntelliCare: a publicly available suite of mental health and well-being apps. Internet Interv.

[18] Harris PA, Taylor R, Thielke R, Payne J, Gonzalez N, Conde JG (2009). Research electronic data capture (REDCap)--a metadata-driven methodology and workflow process for providing translational research informatics support. J Biomed Inform.

[19] Ramo DE, Prochaska JJ (2012). Broad reach and targeted recruitment using Facebook for an online survey of young adult substance use. J Med Internet Res.

[20] Thornton L, Batterham PJ, Fassnacht DB, Kay-Lambkin F, Calear AL, Hunt S (2016). Recruiting for health, medical or psychosocial research using Facebook: systematic review. Internet Interv.

[21] Glickman SW, McHutchison JG, Peterson ED, Cairns CB, Harrington RA, Califf RM, Schulman KA (2009). Ethical and scientific implications of the globalization of clinical research. N Engl J Med.

[22] Mohr DC, Lyon AR, Lattie EG, Reddy M, Schueller SM (2017). Accelerating digital mental health research from early design and creation to successful implementation and sustainment. J Med Internet Res.

[23] Curran GM, Bauer M, Mittman B, Pyne JM, Stetler C (2012). Effectiveness-implementation hybrid designs: combining elements of clinical effectiveness and implementation research to enhance public health impact. Med Care.

[24] Peters-Lawrence MH, Bell MC, Hsu LL, Osunkwo I, Seaman P, Blackwood M, Guillaume E, Bellevue R, Krishnamurti L, Smith WR, Dampier CD, Minniti CP, Sickle Cell Disease Clinical Research Network (SCDCRN) (2012). Clinical trial implementation and recruitment: lessons learned from the early closure of a randomized clinical trial. Contemp Clin Trials.

[25] Sumi E, Teramukai S, Yamamoto K, Satoh M, Yamanaka K, Yokode M (2013). The correlation between the number of eligible patients in routine clinical practice and the low recruitment level in clinical trials: a retrospective study using electronic medical records. Trials.

[26] Huang G, Bull J, Johnston McKee K, Mahon E, Harper B, Roberts JN (2018). Clinical trials recruitment planning: a proposed framework from the Clinical Trials Transformation Initiative. Contemp Clin Trials.

