# Supplementary material for: A Practical Do-It-Yourself Recruitment Framework for Concurrent eHealth Clinical Trials: Identification of Efficient and Cost-Effective Methods for Decision Making (Part 2)
Source: J Med Internet Res. 2018 Nov 29;20(11):e11050. doi: 10.2196/11050 (PMC6293245; doi:10.2196/11050)

Supplemental File 1. Examples of Recruitment Advertisements

Recruitment Site: Craigslist

Northwestern University Depression Research Study

Researchers at the Department of Preventive Medicine at Northwestern University are looking to develop new methods to treat depression through the TeleHealth Study.

We are looking for participants who meet the following criteria:
- Must be over 18 years of age
- Have felt sad or down, or experienced loss of interest and motivation for doing things, for the past 2 weeks or longer.
- Have access to a high speed internet connect

Studies show that people who are depressed may benefit from counseling. This study is examining different ways of providing counseling for depression. If you have been experiencing symptoms of depression, you may be eligible to participate in this research study.

You would be randomly assigned to receive either:
Up to 20 sessions of telephone counseling from a trained clinician
OR
Access to a web based intervention for depression, with regular support calls and emails from a trained clinician (people in this group may be stepped-up to receive 50 minute telephone sessions).

We may conduct five telephone interviews and questionnaires with you during your participation in the study with compensation amounts that increase across time.

Please take our brief eligibility survey if you are interested in participating. Click Here: (link to pre-screener)

You may also contact study staff at 855-682-2487 (855-NUCBITS) for more information.

This study has been approved by the Northwestern University Institutional Review Board (STU00064411)

Recruitment Site: Reddit

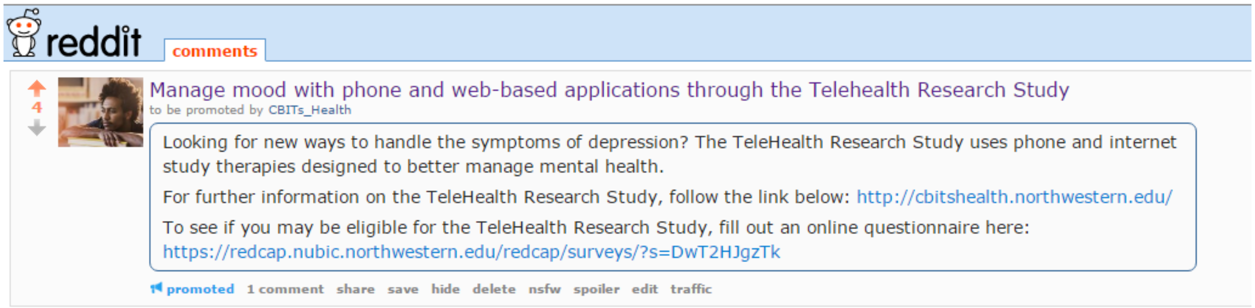

Recruitment Site: Instagram

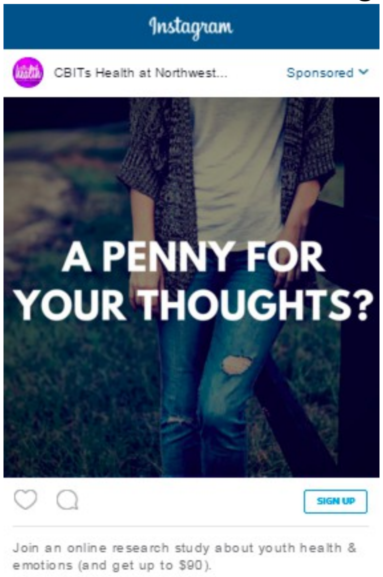

Recruitment Site: ResearchMatch.org

Researchers at Northwestern University's Center for Behavioral Intervention Technologies are conducting a study to evaluate IntelliCare, a suite of mobile phone apps that teach mood management skills to people experiencing depression and anxiety. The apps work to target common issues related to depression and anxiety, like feeling down, stressed or worried.

Expected Commitment:

If enrolled, you will be asked to download the IntelliCare apps onto your own Android smartphone, and use the apps each day for an 8-week period.

You will be randomly assigned to either

Use the apps independently Or Communicate via phone and through text messages with an IntelliCare coach who will help with the use of the apps

Individuals will participate in the 8 week IntelliCare program, during which, two online assessments will be completed. Follow-up online assessments will be conducted 3 and 6 months after the 8 week program. In sum, the IntelliCare study lasts up to 8 months.

Compensation:

Participants will be compensated up to $160 for completing the study assessments.

Eligibility:

This study is for individuals who:

- Have an Android smartphone with a data/text messaging plan
- Are US Citizens or Residents
- Are at least 18 years old
- Are currently experiencing symptoms of depression or anxiety
- Have not previously used the IntelliCare apps

If you are interested in learning more about our study, please let us know through ResearchMatch.

Thank you!

Recruitment Site: The Aging Research Registry

Dear Research Registry Member,

We are writing to provide information about a research study that may be of interest to you called the MoodTech Study. The study is being conducted through the Northwestern University Department of Preventive Medicine by David C. Mohr, Ph.D. We are looking for people ages 65 years and older who have access to a computer, mobile phone, or tablet with an Internet connection AND who have been experiencing symptoms of depression, including:

- Experiencing a loss of interest or pleasure in doing things, feeling sad or experiencing a depressed mood, decreased energy or fatigue, changes in sleep and appetite, feelings of worthlessness and guilt, and trouble concentrating.

If you are 65 years or older and have been experiencing these symptoms, you may be eligible to participate in this research study. Participation in this study is compensated.

This study is evaluating an 8-week online program that aims to help adults learn strategies to reduce depression and improve their mood and quality of life. All eligible participants will receive access to the interactive website and will be assigned to receive one of two versions of the online program: one with a social networking component, and one without. Brief coaching will be provided by trained study staff to support participants in using the website.

Participating in this study will help researchers better understand different methods for treating depression in adults 65 years and older and could contribute to making these programs more widely available to people. As part of the study, you would also complete telephone interviews and online questionnaires over the course of 5 months. You would be compensated for the time spent completing the interviews and questionnaires.

If you are interested in learning more, please contact the study office by telephone at 855-682-2487 (855- NUCBITS) or by e-mail at ehealth@northwestern.edu. You may also find out more by visiting our website at http://cbitshealth.northwestern.edu/.

We thank you for taking the time to read this letter, and would be happy to speak with you if you have any questions.

Sincerely,

David C. Mohr, Ph.D. Principle Investigator
Contact Information: 855-682-2487 (855-NUCBITS). ehealth@northwestern.edu
http://cbitshealth.northwestern.edu/

Recruitment Site: Facebook (paid ads)

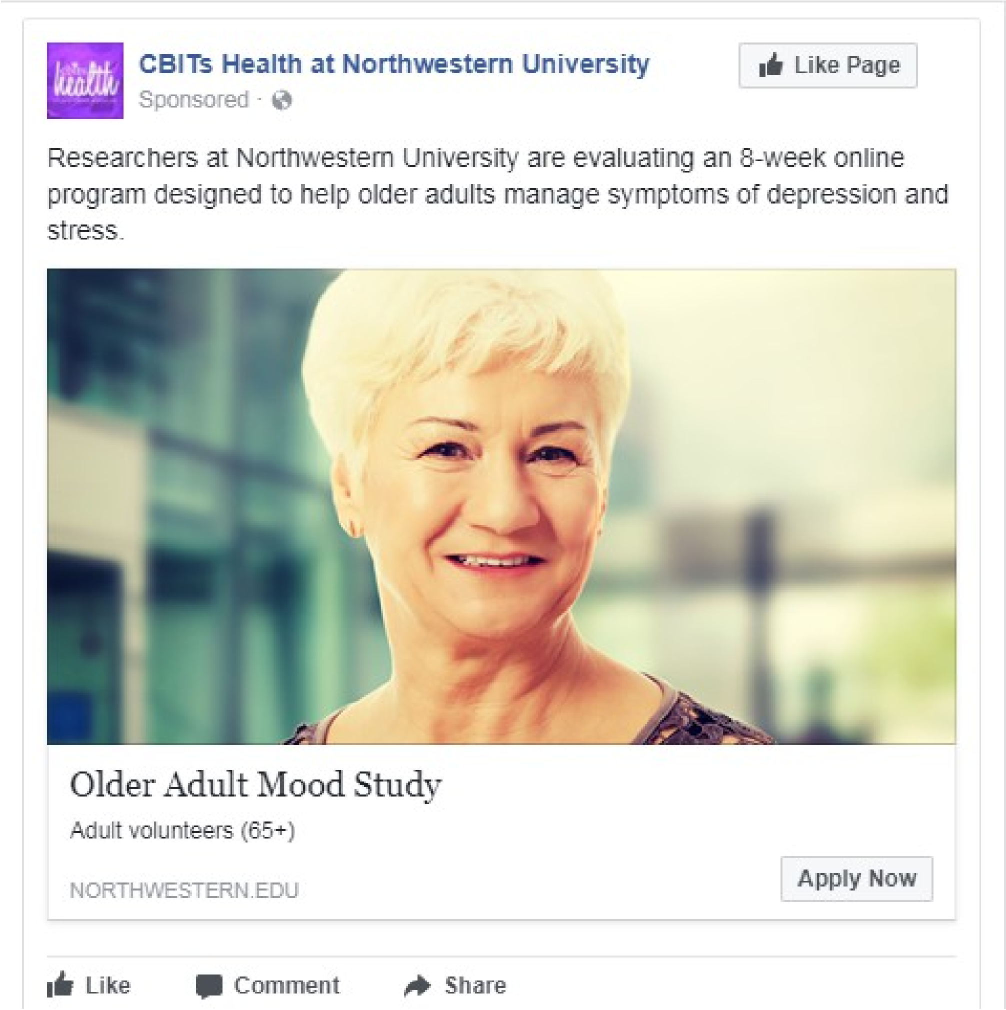

Recruitment Site: Google Ads

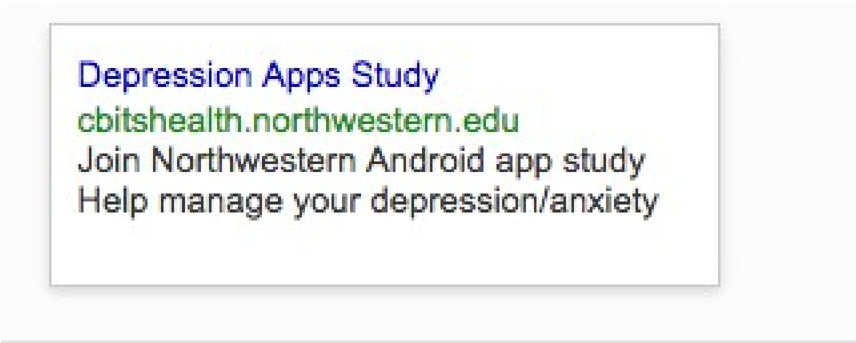

Recruitment Site: DNAinfo Newsletter

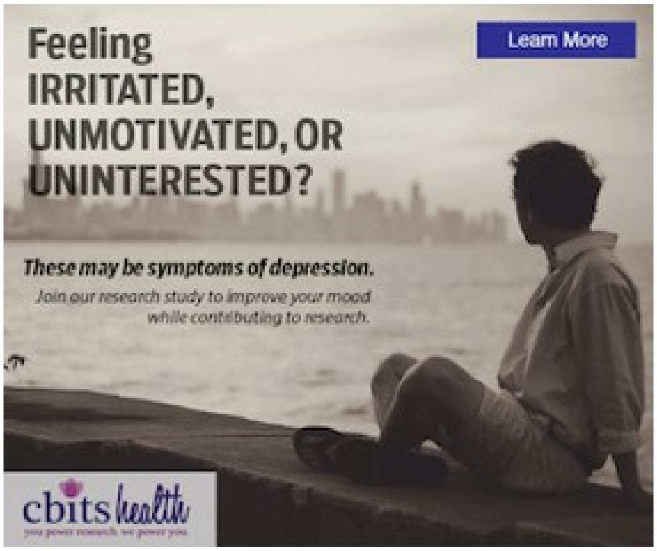

Supplement: Multimedia Appendix 1 [file jmir_v20i11e11050_app1.pdf]
